# Vitamin D Deficiency and Unclear Abdominal Pain in Patients from Low- and Middle-Income Countries

**DOI:** 10.3390/ijerph16234607

**Published:** 2019-11-20

**Authors:** Michael Doulberis, Apostolis Papaefthymiou, Jannis Kountouras, Stergios A. Polyzos, Simone Srivastava, Jolanta Klukowska-Rötzler, Martin Perrig, Sylvana Papoutsi, Aristomenis K. Exadaktylos, David Shiva Srivastava

**Affiliations:** 1 Department of Gastroenterology and Hepatology, Zurich University, 8091 Zurich, Switzerland; 2 Second Medical Clinic, Faculty of Medicine, Ippokration Hospital, Aristotle University of Thessaloniki, 54642 Thessaloniki, Macedonia, Greece; appapaef@hotmail.com (A.P.); jannis@auth.gr (J.K.); 3 Emergency Department, University Hospital Inselspital, 3010 Bern, Switzerland; jolanta.klukowska-roetzler@insel.ch (J.K.-R.); aristomenis.exadaktylos@insel.ch (A.K.E.); davidshiva.srivastava@insel.ch (D.S.S.); 4 Department of Gastroenterology, 401 General Military Hospital of Athens, 11525 Athens, Greece; 5 First Department of Pharmacology, Faculty of Medicine, Aristotle University of Thessaloniki, 54124 Thessaloniki, Macedonia, Greece; spolyzos@auth.gr; 6 Department of Visceral Medicine and Surgery, University Hospital Inselspital, 3010 Bern, Switzerland; simone.srivastava@insel.ch (S.S.); sylvana.papoutsi@insel.ch (S.P.); 7 Department of General Internal Medicine, University Hospital Inselspital Bern, 3010 Bern, Switzerland; martin.perrig@insel.ch

**Keywords:** emergency department, abdominal pain, vitamin D, low income, middle income, migrants, irritable bowel syndrome (IBS)

## Abstract

**Background:** Abdominal pain is one of the commonest symptoms in emergency departments (EDs). Diagnosis demands full attention and critical thinking, since many diseases manifest atypically and the consequences of overlooking the symptoms may be disastrous. Despite intensive diagnostic procedures, some cases remain elusive and unclear abdominal pain (UAP) is not infrequent. Emerging evidence supports the hypothesis that functional pain might be attributed to vitamin D deficiency (VDD). People with darker or covered skin are predisposed to developing VDD. Patients in Switzerland stemming from low- and middle-income countries (LMIC) are such a population. **Aim:** To identify cases with UAP in LMIC patients and to compare vitamin D status with a control group. **Methods:** A retrospective single-center case-control study was carried out from 1 January 2013 to 31 August 2016 in all adult patients (more than 16 years old) stemming from LMIC and presenting at the university ED of Bern with abdominal pain. Vitamin D status was retrieved from these cases when available. The control group consisted of patients without abdominal pain or metabolic diseases and was matched (1:1) to the cases for age, gender, body mass index, geographic distribution, and season of vitamin D estimation. **Results:** A total of 10,308 cases from LMIC were reported to the ED. In total, 223 cases were identified with UAP. The status of vitamin D was available for 27 patients; 27 matched individuals were subsequently retrieved for the control group. Women made up 56.7% of the UAP group and 43.3% of the control group. The most common origin of the LMIC subjects was southern Europe (20.4%), followed by southern Asia (16.7%) and Eastern Europe (13%). Fourteen UAP patients exhibited severe VDD (<25 nmol/L) versus one in the control group (*p* = 0.001). The difference remained significant if the patients were identified as having VDD (<50 nmol/L) or not (*p* = 0.024). Comparison of the means indicated that the UAP group had lower vitamin D levels than the control group (41.3 vs. 53.7 nmol/L, respectively), but this difference was marginal (*p* = 0.060) and not statistically significant. After adjustment for potential confounders, including gender, mean vitamin D levels remained non-significantly different between groups. In the sub-group analysis, vitamin D levels were lower in women than in men (*p* = 0.037), compared to the respective controls. **Conclusion:** This study showed for the first time that patients from LMIC who presented to ED with UAP displayed VDD. Validation from larger studies is warranted to evaluate the linkage of VDD with UAP.

## 1. Introduction

The definition of pain as given by the International Association for the Study of Pain is “an unpleasant sensory and emotional experience associated with actual or potential tissue damage, or described in terms of such damage” [1]; it is also described as “a distressing experience associated with the mentioned actual or potential tissue damage with sensory, emotional, cognitive and social components” [2]. Abdominal pain (AP) may be acute or chronic. Since a uniform terminology is required in patients with acute AP, in order to clarify findings and to facilitate comparison between different studies, the following definition for acute AP has been introduced: “Pain of non-traumatic source with a maximum length of 5 days” [3]. Chronic AP, although frequent, is difficult to define. The predisposing factors are inadequately understood and the pathogenesis is unclear; the prevailing opinion in pathophysiology emphasizes the inter-relationship between alterations in hypersensitivity and changed motility, with many associated risk factors [4].

Acute AP is one of the commonest causes of admission to the emergency department (ED) of tertiary hospitals [5,6,7,8]. AP can have several medical and surgical causes, both gastrointestinal (GI) and non-GI, and its assessment and management in the ED should be rapid and accurate [4]. Diagnostic procedures may be challenging, even for experienced clinicians, since the range of differential diagnosis is wide and a combination of history, radiological, clinical as well as laboratory evaluation is mandatory. Furthermore, any delay in diagnosis and potential initiation of treatment might influence the outcome and prognosis [5,8,9]. Special populations like the elderly, immunocompromised patients [5] and patients with foreign linguistic and cultural backgrounds [10] demand additional attention, since the clinical presentation and history may be atypical or even misleading and this may give rise to misdiagnosis of abdominal pathologies.

In recent decades, a continuously increasing number of migrants and refugees from low- and middle-income countries (LMIC) have arrived in Europe to strive for a better future [11]. Moreover, there has been a substantial increase in admissions to EDs of LMIC patients claiming symptoms of acute AP [12,13]. The different cultural, genetic, linguistic as well as sanitary background of these patients from LMIC—or poorer countries—has an impact on the different stratification of pathologies and percentages that are seen in the ED and this tendency influences the sociodemographic structures, including EDs [14].

Among European countries, Switzerland has the highest rates of permanent foreigners. In 2017, 2,108,001 migrants (24.8%) were documented according to the Swiss Federal Statistical Office [12]; this must be compared with Europe’s mean migrant contingent of 7.3% to 8.6%, and Switzerland supports the highest contingent of foreign population, with 23.5% [15]. Psychological stress, self-medication (laxatives, cocaine), sexual abuse and even uncommon infectious diseases (e.g. tuberculosis, human immunodeficiency virus, histoplasmosis and others) are frequent causes of AP in migrants [15]. Immigration-specific features are also evident in the interpretation of symptoms: Not every “AP” is really AP and, thus, it is critical to make the distinction between organic, functional, and psychological AP. This distinction can be in the setting of the patient’s overall situation and the clinician may need help from members of the same cultural group or a skilled interpreter.

Recent evidence supports the concept that functional AP, for instance, irritable bowel syndrome (IBS), might be attributed to vitamin D deficiency (VDD), particularly when there is no anatomical–organic correlation or other explanatory pathology [16,17,18,19,20]; VDD is associated with more severe AP, increased scores for the severity of IBS symptoms, and lower quality of life in IBS patients [21]. Moreover, the specific religious or stylistic habits of Muslim patients [22] as well as patients of African [23,24] and middle East origin [25,26] may have an impact on the exposure of skin to the sun and may explain why these patients have a well-documented predisposition to developing VDD [22,23,24,25]. Therefore, it would be of interest to investigate whether unclear AP (UAP) is associated with VDD.

The main aim of our retrospective case-control study was to investigate VDD in patients presenting with UAP to the ED of Bern University Hospital (commonly known as the Inselspital). The secondary aim was to investigate whether demographic parameters affect the potential association between vitamin D status and UAP.

## 2. Materials and Methods

### 2.1. Study Design

We performed a retrospective single-center case-control study including patients presenting at the ED of Inselspital Bern with symptoms or/and signs of AP from 1 January 2013 to 31 August 2016. Inselspital ED serves both as a tertiary referral center (Level 1) and primary care facility, and provides medical services to an area of about two million citizens; in 2017, approximately 46,000 adult patients were admitted as emergencies to the ED [27].

#### Inclusion and Exclusion Criteria

We included patients aged >16 years from LMIC who presented to the ED with UAP. Exclusion criteria were the presence of structural abdominal pathologies, such as pancreatitis, cholecystitis, cholangitis, acute hepatitis, and bowel obstruction (ileus); urological/renal disorders (including infection, lithiasis); gynecological, oncological, or rheumatological disorders; transplantation (heart, kidney, liver, lungs); alcohol/drug intoxication; presence of postoperative abdominal adhesions; acute coronary syndrome; and patients with elevated inflammation markers (C-Reactive protein, CRP, erythrocyte sedimentation rate, ESR, leucocytes) and/or fever, suggestive of acute infection. Moreover, patients taking vitamin D or calcium supplements were not included as well as pregnant women and patients with psychosis. Patients were also excluded if they had not been subjected to additional necessary examinations.

For the control group, we recruited participants from the same sampling pool (LMIC), with available vitamin D status, without abdominal pain, who presented with panic attacks and/or palpitations in ED. The controls were matched (1:1) with cases for age, gender, and body mass index (BMI). The exclusion criteria for the control group were the same as for the patients.

### 2.2. Data Collection and Extraction

Records of patients upon admission to the ED are registered in the clinical application E.care for Windows (E.care BVBA, ED 2.1.3.0, Turnhout, Belgium). This medical database enables instantaneous recall of past diagnostic reports, consultations, X-rays, and other relevant medical documents. Additionally, multiple filters of E.care database were applied, including time frame, age, medical/surgical cases, and LMIC. Country of origin along with information on resident status were retrieved by the hospital administration and recorded in the hospital information system (SAP).

The following parameters were extracted from the charts of included patients: Demographics (age, gender), type of transport to the ED (e.g., by ambulance), triage, date of admission and discharge, medical history, symptoms and their duration, laboratory findings, radiological findings (including abdominal computer tomography and/or ultrasound, where needed), treatment provided, type of discharge from the ED (e.g., inpatient admission, outpatient therapy), length of hospital stay, and outcome (e.g., complications, mortality).

Patients were routinely triaged in the Inselspital by using the Swiss Emergency Triage Scale [28]. The latter is an abbreviated version of the validated Manchester Triage System [29]. This triage system stratifies the urgency of treatment for patients presenting to an ED by the five following levels: 1: Acute life threating problem (immediate treatment required); 2: High urgency; 3: Urgency; 4: Less urgency; and 5: No urgency. Once a new patient had been admitted to the ED, a specially trained nurse assigned the patient’s reported complaints according to a defined algorithm and then determined the treatment priority with the aid of fixed rules that also consider the vital signs.

**Figure 1:**
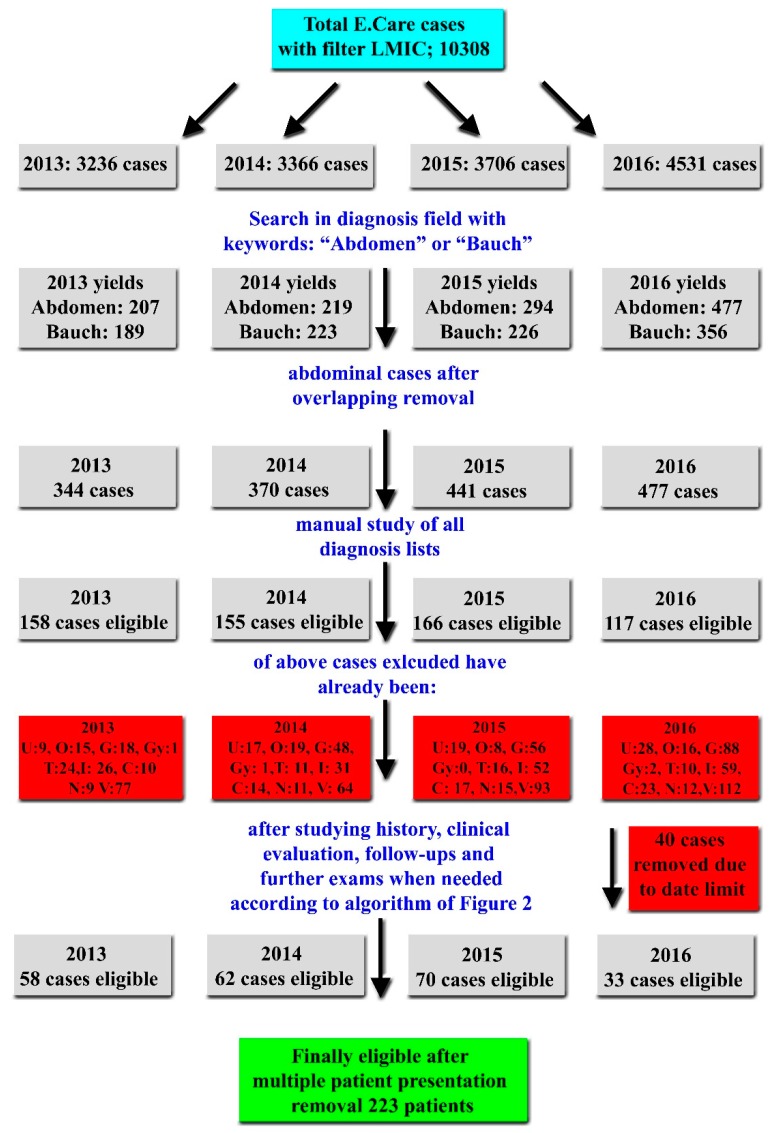
Flow chart of the search procedure for the identification of eligible patients. C; cardiological cases, G; gastrointestinal cases, Gy; gynecological cases; I; infectious cases, LMIC; low- and middle-income countries, N; neurological cases; U; urological/renal cases, Trauma cases, V; various cases (such as; body packer, intoxications (including alcohol), suicide attempt, rheumatological, ORL, unclear thoracic pain-dyspnea, orthopedic without trauma, thrombosis etc.).

**Figure 2:**
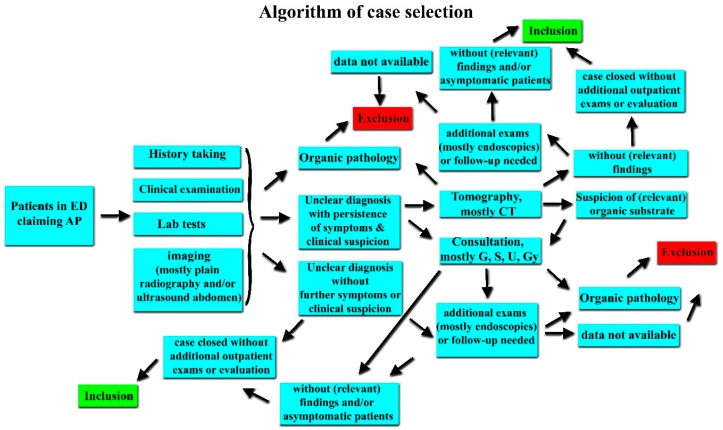
Schematic representation of the chosen algorithm for case selection. AP; abdominal pain, ED; emergency department, CT; computer tomography, G; gastroenterologist, Gy; gynecologist, S; surgeon, U; urologist/nephrologist.

All patients who presented to the ED during the mentioned time frame were extracted from E.Care to an Excel sheet (Microsoft^®^ Excel for Mac 2019, Microsoft Corporation, Redmond, WA, US) for further analysis. Patients with AP were identified by using the following search strings in the patient “diagnosis list” column: “Abdomen” (also in German) and “Bauch” (in English translated colloquially as “belly”). The resulting cases were further manually investigated by two authors (M.D. and A.P.), a s explained in Figure 1 and Figure 2. An in-depth manual investigation was performed by M.D. with the retrieval of detailed procedures when necessary, such as radiological or endoscopic findings. Validation of the extracted data was performed by D.S.S. In cases of disagreement, a consensus was achieved by the intervention of a senior author (A.K.E.).

### 2.3. Definition of Economies

According to the World Bank [30] for the current 2019 fiscal year, “low-income economies were defined as those with a gross national income (GNI), calculated using the World Bank Atlas method, of $995 or less in 2017; lower middle-income economies were included those with a GNI per capita between $996 and $3895; upper middle-income economies were those with a GNI per capita between $3896 and $12,055; and high-income economies were those with a GNI per capita of $12,056 or more”.

The countries of origin were grouped as defined by the origin classification of the United Nations [31].

### 2.4. Vitamin D Estimation

The serum levels of 25-OH-cholecalciferol (25OHD) were measured by certified commercial laboratories using liquid chromatography-mass spectrometry and the MassChrom® 25-OH-Vitamin D3/D2 diagnostics kit (ChromSystems, Munich, Germany). The intra-assay coefficient of variation (CV) was <5% and interassay CV was <6%. In accordance with previous guidelines for the definition of vitamin D status, calcifediol concentrations <50 nmol/L were defined as VDD [32]. Severe VDD was defined as concentration <25nmol/L. On 1 September 2016, the vitamin D analysis was switched from liquid chromatography-tandem mass spectrometry to a chemiluminescence immunoassay. In order to enhance homogeneity and to facilitate the interpretation, we included cases and controls with measured 25OHD up to 31 August 2016. If multiple values of vitamin D were found, the first value was taken, as this was closest to UAP.

### 2.5. Ethical Considerations

This descriptive retrospective study was carried out in compliance with the last revision of the principles of the Declaration of Helsinki [33] as well as Swiss law and was approved by the cantonal (district) ethics committee in Berne (Kantonale Ethikkommission Bern, Ref. No. KEK-BE: 010/ 2016). According to the ethics committee, written informed consent was unnecessary, due to the retrospective nature of the study and the fact that our cases and controls were completely anonymized.

### 2.6. Statistical Analysis

Data are presented as mean ± standard error of the mean (SEM) or as frequencies, for continuous or categorical variables, respectively. The normality of distributions of continuous variables was checked by the Kolmogorov–Smirnov test. A comparison of continuous variables was performed with independent samples, using the t test or the Mann–Whitney test. A comparison of categorical variables was performed with the chi-square test or the Fischer exact test. Analysis of covariance (ANCOVA) was performed to adjust between group comparisons for potential cofounders. Statistical analysis was performed with SPSS 21.0 for Macintosh (IBM Corp., Armonk, NY). Significance was set at *p* < 0.05 (two tailed).

## 3. Results

During the period 2013–2016, a total of 10,308 cases from LMIC were reported in the ED of Inselspital. After applying the algorithm described in detail in Figure 1 and Figure 2, a total of 223 cases were identified with UAP. Of these, 27 patients had available data of vitamin D status in our records. Seventeen of these were women (Table 1). A control group of 27 individuals of similar gender, age, and BMI were enrolled (Table 2). Fourteen UAP patients had severe VDD (defined as <25 nmol/L), whereas only one participant from the control group exhibited severe VDD. This difference was statistically significant (*p* = 0.001, Table 3). If the cut-off value for VDD was raised to 50 nmol/L), the difference between UAP patients and the control group remained significant (*p* = 0.024, Table 3).

However, UAP patients had mean levels of 25OHD similar to the control group (41.26 vs. 53.67 nmol/L, respectively), and the difference just failed to reach statistical significance (*p* = 0.060) (Table 2).

**Figure 3:**
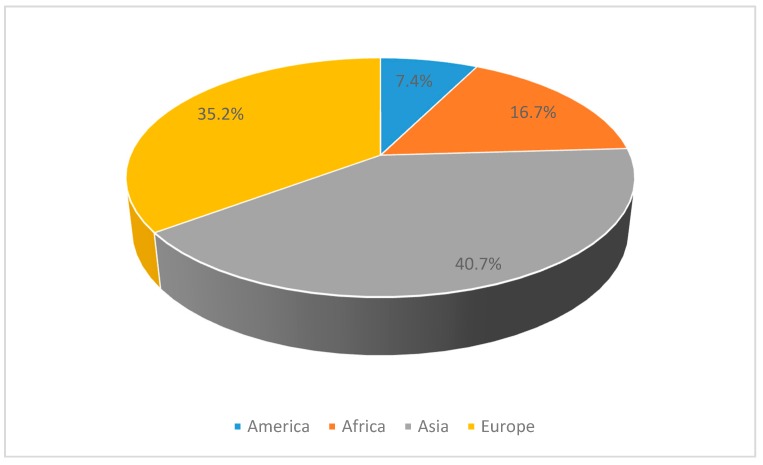
Continental distribution of subjects.

**Figure 4:**
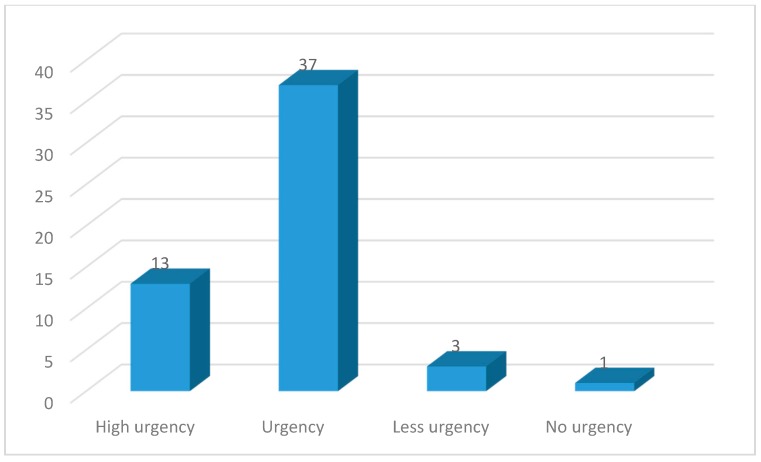
Swiss Emergency Triage Scale depicting the number of cases per category.

An attempt was made to identify the region of origin for all participants (n = 54; Table 4). The most common region of origin was Southern Europe (20.4%), followed by Southern Asia (16.7%) and Eastern Europe (13%). The continental distribution is depicted in Figure 3. Within the triage system of Inselspital, most cases (37) were labeled as urgent, whereas only one case was non-urgent (Figure 4). For LMIC patient in both groups, the most common form of admission was self-admission and the most common form of discharge was at home (Table 5).

In the sub-group analysis, there was no difference between the two groups regarding the season of blood collection for vitamin D. Likewise, there were no differences in vitamin D between patients and controls with respect to continent of origin, triage, or manner of admission (Supplement). In a further separate sub-group analysis in men and women, 25OHD levels were significantly lower in women with UAP than in women without UAP (*p* = 0.037, Table 6). In contrast, there were no significant differences between 25OHD levels in men with and without UAP.

## 4. Discussion

To the best of our knowledge, this study showed for the first time that patients from LMIC who presented to ED with UAP had an increased prevalence of VDD. In general, pain of a functional nature, without any evidence of organic etiology, may be a manifestation of IBS. In this regard, abdominal pain associated with functional GI disorders occurs in a significant percentage of children and adolescents referred to gastroenterology clinics or ED; these disorders include IBS, migraine, functional dyspepsia, or psychiatric disorders [34]. Moreover, IBS appears to be a source of considerable burden and cost in the United States [35]. Therefore, management of such patients with VDD might be a cost-effective strategy, although this remains to be shown. This concept is supported by recent publications, as follows.

In a case-control study [19], 60 IBS patients (who fulfilled Rome III diagnostic criteria) were compared to 100 healthy individuals with respect to vitamin D status. It was shown that the IBS group exhibited significantly lower 25OHD levels relative to the control group. Moreover, 112 IBS adults with known VDD were randomly divided in two groups, one receiving placebo and one vitamin D supplementation. When compared with placebo, the group that had received vitamin D for 6 months showed a statistically significant improvement, as expressed by scoring systems [17]. Besides, a relevant experiment was performed by an Iranian research team, where soy isoflavones, vitamin D and placebo were administered in all different combinations to IBS adults and amelioration of severity scores was recorded [18]. In another study [21], 90 IBS patients and the same number of healthy controls were recruited. The IBS group exhibited enhanced incidence of VDD, more severe clinical symptoms, and raised IBS scores. Similar conclusions were drawn by another study group from the USA [16]; in a retrospective study with 55 children with IBS and 100 controls, it was shown that more than half of IBS patients had VDD < 50 nmol/L and >90% of IBS subjects had VDD < 75 nmol/L (93% vs. 75%, *p* = 0.006). Moreover, IBS patients had significantly lower mean 25OHD. In a pediatric ED setting, Kehler et al [36] identified 89 children with VDD, the majority (83%) with severe VDD. The most common clinical manifestation for ED admission was abdominal pain and the most common ethnic origins were black African and Pakistani. In a second UK study, with a randomized, double-blind, three-arm design, placebo, vitamin D and probiotics were administered to IBS patients. The authors deduced that IBS patients demonstrate significantly lower levels of vitamin D and would benefit from screening and possible supplementation.

The impact of IBS on quality of life might be affected by 25OHD levels. Interestingly, VDD is strongly associated with functional problems in intestinal motility, accompanied by psychiatric disorders (anxiety and depression) that severely influence the quality of life of such patients. VDD and symptoms of anxiety and depression are frequently connected with chronic functional constipation provoked by disorders in intestinal motility. 25OHD serum concentrations should be routinely estimated in these patients and vitamin D supplementation may offer better control of intestinal motility and quality of life [37].

Aside from IBS, Whitehurst et al. reported two cases of unexplained chronic diffuse pain of the abdomen, pelvis, or extremities in the palliative setting, where VDD could be confirmed. After the substitution and normalization of vitamin D, both patients claimed that the pain was resolved [38].

Functional pain has also been reported extra-abdominally and associated with VDD. For instance, non-cardiac chest pain was associated with low levels of 25OHD in two studies [39,40]. Additionally, migraine, another pathology without structural substrate, was also associated with VDD. In this respect, in a recent randomized, double-blinded, placebo-controlled trial, supplementation of Vitamin D was shown to be superior to placebo in reducing migraine days [41]. Moreover, in a prospective case-control study, it was demonstrated that migraine had severe VDD compared to the control group and that there was a linear negative relationship between headache days and serum 25OHD levels [42]. Another study showed that a larger number of monthly days with headache was related to VDD among patients with migraine [43]. Lastly, comparable additional data on migraines and VDD has been reported in another case-control study from Iran [44].

Our study has certain limitations. These include the retrospective nature of the design and the relatively small number of subjects with UAP and available vitamin D status. However, this population largely consists of refugees and asylum seekers, so medical compliance is not always warranted and the patients do not stay long term in a single country; in our case, in Switzerland. Therefore, elective follow-ups with optimal care are not always feasible. Additionally, for the purpose of statistical analysis, the assumption that all subjects with vitamin D levels <25 nmol/L have levels equal to 25 nmol/L may have influenced the statistical significance when mean vitamin D levels were compared between patients and controls. Furthermore, the results were not controlled for season. Lastly, a selection bias for the control group has to be acknowledged.

## 5. Conclusions

For the first time, we report higher rates of VDD and severe VDD in patients with UAP than controls. Although the observational nature of our study cannot prove causality, accumulating evidence in the literature warrants mechanistic studies on the potentially contributing role of vitamin D to pain perception.

In our subgroup analysis, female, but not male, patients had significantly lower vitamin D levels than the respective controls, but this difference did not remain robust after adjustment for potential cofounders. Our results warrant studies with larger sample sizes as well as mechanistic studies to investigate the potential sexual dimorphism in vitamin D status in patients with UAP.

Nevertheless, the clinician should be particularly meticulous with patients from other cultural/geographical backgrounds suffering from UAP presented to the ED: Functional abdominal pain is an exclusion diagnosis (clinically, radiologically, interventionally, laboratory analyses). IBS could be a form of such pain. Vitamin D supplementation, when needed, is a simple regimen, which might offer a cost benefit for affected patients, but this remains to be shown.

## Figures and Tables

**Table 1:** Percentages of genders per group.

			Group		Total
			Controls	Patients	
Gender	Women	Count	13	17	30
		% within gender	43.3%	56.7%	100.0%
		% within group	48.1%	63.0%	55.6%
	Men	Count	14	10	24
		% within gender	58.3%	41.7%	100.0%
		% within group	51.9%	37.0%	44.4%
Total		Count	27	27	54
		% within gender	50.0%	50.0%	100.0%
		% within group	100.0%	100.0%	100.0%

**Table 2:** Basic parameters.

	Group	n	Mean	Std. Deviation	Std. Error Mean	*p*-Value
Age	Controls	27	47.70	13.088	2.519	0.085
	Patients	27	41.30	13.708	2.638	
BMI	Controls	27	26.163	5.6835	1.0938	0.186
	Patients	27	24.259	4.7021	0.9049	
25OHD* (nmol/L)	Controls	27	53.67	22.940	4.415	0.060
	Patients	27	41.26	24.513	4.718	

*: 25-OH-cholecalciferol.

**Table 3:** VDD in both groups.

				Group		Total
				Controls	Patients	
25OHD ^a^ nmol/L	≤25		Count	1	14	15
			% within group	3.7%	51.9%	27.8%
	>25		Count	26	13	39
			% within group	96.3%	48.1%	72.2%
Total			Count	27	27	54
			% within group	100.0%	100.0%	100.0%
25OHD ^b^ nmol/L		≤50	Count	13	21	34
			% within group	48.1%	77.8%	63.0%
		>50	Count	14	6	20
			% within group	51.9%	22.2%	37.0%
Total			Count	27	27	54
			% within group	100.0%	100.0%	100.0%

^a^ *p* < 0.001; ^b^ *p* = 0.024; 25OHD: 25-OH-cholecalciferol; VDD: vitamin D deficiency.

**Table 4:** Geographical regions of origin for LMIC(low- and middle-income countries) subjects.

	Frequency	Percent	Valid Percent	Cumulative Percent
South America	3	5.6	5.6	5.6
Eastern Africa	3	5.6	5.6	11.1
Northern Africa	6	11.1	11.1	22.2
Caribbean	1	1.9	1.9	24.1
Eastern Asia	5	9.3	9.3	33.3
Southern Asia	9	16.7	16.7	50.0
South-eastern Asia	2	3.7	3.7	53.7
Southern Europe	11	20.4	20.4	74.1
Western Asia	6	11.1	11.1	85.2
Eastern Europe	7	13.0	13.0	98.1
Northern Europe	1	1.9	1.9	100.0
Total	54	100.0	100.0	

**Table 5:** Types of admission and discharge for LMIC subjects.

		Frequency	Percent	Valid Percent	Cumulative Percent
**Admission**	Police	1	1.9	1.9	1.9
	Self-admission	38	70.4	70.4	72.2
	Ambulance	5	9.3	9.3	81.5
	Private (GP)	3	5.6	5.6	87.0
	Primary utilization	3	5.6	5.6	92.6
	Other or not specified	4	7.4	7.4	100.0
	Total	54	100.0	100.0	
Discharge	At home	42	77.8	77.8	77.8
	Inpatient	9	16.7	16.7	94.4
	Other or not specified	3	5.6	5.6	100.0
	Total	54	100.0	100.0	

GP: general practitioner; LMIC: low- and middle-income countries.

**Table 6:** Sub-group analysis for women.

	Group	n	Mean	Std. Deviation	Std. Error Mean	*p*-Value
Age	Controls	13	43.31	13.073	3.626	0.179
	Patients	17	36.65	13.124	3.183	
BMI	Controls	13	26.908	5.8273	1.6162	0.194
	Patients	17	24.329	4.7864	1.1609	
25OHD (nmol/L)	Controls	13	53.31	25.316	7.021	0.037
	Patients	17	36.59	16.382	3.973	

25OHD: 25-OH-cholecalciferol.

## STROBE Statement

This study has been revised according to the STROBE checklist.
